# Pollen food allergy syndrome secondary to molds and raw mushroom cross-reactivity: a case report

**DOI:** 10.1186/s13223-023-00865-5

**Published:** 2024-01-04

**Authors:** Ryan Gauld, Graham Walter, Rongbo Zhu

**Affiliations:** Division of Clinical Immunology and Allergy, Department of Medicine, Western University, London, ON Canada

**Keywords:** Pollen food allergy syndrome, Cross-reactivity, Mold aeroallergens, Edible fungi, Sensitization

## Abstract

**Background:**

Pollen food allergy syndrome (PFAS) is an immunoglobulin E (IgE) mediated reaction that causes oropharyngeal pruritus or angioedema due to homologous proteins present in the culprit food as well as a sensitizing aeroallergen. This cross-reactivity has been well established between pollen and fruits/vegetables. Given the evolutionary similarity between all fungi; cross-reactivity between spore forming microfungi and edible macrofungi have been suggested, however only a limited number of case reports have ever been published on this phenomenon. We present a case of a patient who experiences pollen food allergy syndrome-like symptoms following lightly cooked mushroom ingestion who otherwise was able to tolerate cooked mushrooms. We then review the literature to highlight the limited studies of an underrecognized PFAS cross-reactivity between molds and mushrooms.

**Case presentation:**

A 15-year-old male presents with symptoms of seasonal and perennial allergic rhinitis was found to have multiple environmental sensitizations to molds via skin prick testing (*C.* *gramineum*, *A.* *Pullulans* and *B.* *cinerea*) and ImmunoCAP serum-specific IgE (*A.* *alternata*, *C. herbarum*, and *P. notatum*). He developed throat pruritus and subjective throat tightness following ingestion of mushroom containing pizza. ImmunoCAP serum specific IgE to whole mushroom was negative but fresh food prick testing to fresh portobello mushroom and cremini mushroom were both positive with a negative test to canned mushroom. The patient then underwent a graded oral challenge and successfully tolerated canned mushrooms.

**Conclusion:**

This case highlights the potential cross-reactivity between microfungi aeroallergens and edible fungi, leading to PFAS-like reactions in susceptible individuals. The patient’s ability to tolerate canned mushrooms suggests a possible heat-labile protein as the cause of the reaction, similar to PFAS patients tolerating cooked but not raw fruits/vegetables. Positive skin prick test to both spore-forming fungi and edible fungi with negative and whole mushroom IgE results further support the hypothesis of cross-reactivity and sensitization. Further research is needed to identify the specific allergenic proteins involved in these cross-reactions and the susceptible species of mold and mushroom. Understanding these components will contribute to improved diagnosis and management of mold and mushroom allergies, and enhance our knowledge of allergenic cross-reactivity in general.

## Background

Fungi are eukaryotic non-chlorophyllous heterotrophic organisms with over 100,000 known species [1]. Molds are microfungi that are ubiquitous in the environment, with allergic implications due to production of airborne spores perennially. Allergies to mold can manifest in different conditions including but not limited to allergic rhinitis, allergic asthma, atopic dermatitis, allergic bronchopulmonary aspergillosis, allergic sinusitis, and hypersensitivity pneumonitis [1]. Mold allergies range from 6–24% in the general population, 44% in atopics, and 80% in asthmatics, and are responsible for the majority of fungal allergies [1]. Conversely, mushrooms are edible macrofungi which can rarely cause anaphylaxis when ingested with only a small number case reports described [2].

All edible fungi fall within the taxonomy divisions *Basidiomycete*s and *Ascomycetes* [3]. Given evolutionary similarity between all fungi, proteins produced by edible fungi can share immunological similarities to microfungi resulting in cross-reactivity. This cross-reactivity is akin to the pollen/fruit/vegetable cross-reactivity seen in pollen food allergy syndrome (PFAS) [1]. This is believed to be an immunoglobulin E (IgE) mediated phenomenon that typically presents with oropharyngeal pruritis, and in some cases angioedema due to homologous proteins present in the culprit food as well as the sensitizing aeroallergen [4]. In general, immunological similarity between epitopes occurs between closely related taxonomical species or between epitopes with similar function belonging to the same protein family [4, 5].

Exposure to epitopes of aeroallergens via the respiratory tract can cause sensitization that leads to symptoms with first exposure to similar epitopes within food. Cross-reactivity between several pollens and foods have been well reported [6]. Cross-reactivity between spore forming fungi and mushrooms has been suggested, however few case reports have ever been described in the literature [7]. Herein, we describe a case of a young male experiencing PFAS symptoms following mushroom ingestion who is able to tolerate mushroom in more extensively processed form.

## Case presentation

A 15-year-old-male with a history of mild atopic dermatitis, peanut, and tree nut allergy previously diagnosed via skin prick testing (SPT) by another Allergist. He was referred to a hospital-based allergy clinic for asthma management. In addition to dyspnea and wheeze, he suffered from symptoms of seasonal and perennial allergic rhinoconjunctivitis. Skin prick testing to ALK allergen extracts was positive to grass, ragweed, *Botrytis cinerea*, *Cephalosporium gramineum*, and *Aureobasidium* *pullulans* with appropriate positive 1 mg/ml histamine and negative saline controls (Table 1); with positive testing defined as a wheal with largest diameter ≥ 3 mm larger than the negative control. He was prescribed ciclesonide nasal spray 50 mcg 1 spray twice daily to each nostril with proper technique reviewed.

**Table 1:** Summary of skin prick test (SPT) and serum specific immunoglobulin E (IgE) results

Aeroallergen SPT	Result	Food SPT	Result	IgE	Result
Grass	+	Portobello	5 mm	*Alternaria alternata*	3.56 kU/l
Ragweed	+	White Cremini	6 mm	*Cladosporium herbarum*	2.28 kU/l
Botrytis cinerea	+	Brown Cremini	4 mm	*Penicillium notatum*	19.45 kU/l
Cephalosporium gramineum	+	Canned	Negative	*Agaricus hortensis*	< 0.35 kU/l
Aureobasidium pullulans	+			Total	764 kU/l

Weeks following this assessment, the patient ingested a pizza with fresh mushrooms (*Agaricus bisporus*), and developed immediate throat pruritis and subjective throat tightness. He had ingested edible fungi previously without any issue or symptom. He was certain there was no cross-contamination with peanuts or tree nuts.

ImmunoCap serum specific IgE (from Phadia Laboratory Systems) were positive for *Alternaria alternata* (3.56 kU/l), *Cladosporium herbarum* (2.28 kU/l), and *Penicillium notatum* (19.45 kU/l); with a positive test defined as ≥ 0.35 kU/l. ImmunoCap serum specific IgE to whole mushroom (*Agaricus hortensis*) was negative with total IgE 764 kU/l (Table 1).

**Fig. 1:**
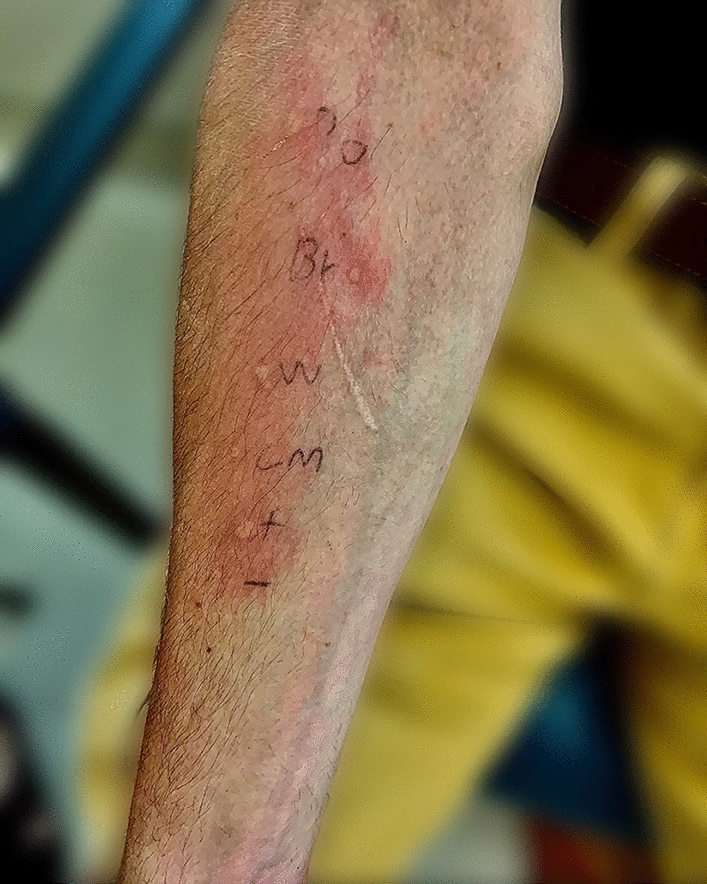
Skin prick test to Portobello mushroom (PO), White Cremini mushroom (W), Brown Cremini mushroom (Br), and canned mushroom (CM) with appropriate positive histamine (+) and negative saline (−) controls

Subsequent SPT to fresh mushroom was positive for portobello mushroom (5 mm wheal), white cremini mushroom (6 mm wheal), and brown cremini mushroom (4 mm wheal). It was also negative to canned mushroom with an appropriate positive 1 mg/ml histamine and negative saline controls (Table 1, Fig. 1). Given these results, the patient underwent a 5-step graded oral challenge to canned mushrooms to a cumulative 3 g of mushroom protein as defined by the ingredient label. He successfully tolerated the challenge without any oral pruritis or other systemic symptoms suggestive of anaphylaxis. The patient declined an oral challenge to raw mushrooms to fully confirm PFAS as he did not want to experience recurrent clinical symptoms. He was already equipped with an epinephrine autoinjector due to pre-existing allergies and was advised to avoid consumption of raw or undercooked mushrooms as a precautionary measure.

## Discussion and conclusion

Wide-spread cross-reactivity between the phyla *Basidiomycete*s and *Ascomycetes* has been well established [1]. However, only a few cases of PFAS-like reaction from mushroom cross-reactivity have ever been reported [4]. Dauby et al. (2002) described the first case of PFAS with uncooked mushroom in a patient allergic to molds. They showed a cross-reactivity between raw heat labile mushroom proteins of 43 kilodaltons (kD) and 67 kD and molds *Horodendrum cladosporioides*,* Alternaria tenuis*,* Fusarium vasinfectum*, and *Helminthosporium interseminatum* [8]. Another case report with PFAS to mushroom and anaphylaxis to spinach showed cross-reactivity between epitopes from the mold species *Alternaria alternata* and *Cladosporium herbarum*, and epitopes from the mushroom *Agaricus bisporus* and spinach [9]. Glabriel et al. [7] reported a patient who suffered anaphylaxis postulated to be secondary to cross-reactive IgE between mold aeroallergens and thermostable proteins found in *Agaricus bisporus*. The proteins were identified to be a 24 kD manganese-dependent superoxide dismutase (MnSOD) and 27 kD NADP-dependent mannitol dehydrogenase [7]. Most recently, Betancor et al. [10] identified a heat stabile cross-reactive protein between *Agaricus bisporus* and *A. alternata* identified to be a 36 kD member of the porin family.

Our case adds to the developing body of knowledge by describing a patient with a history of allergic rhinitis to microfungi aeroallergens who developed PFAS-like reaction with mushroom ingestion. Despite this reaction, they were able to pass a graded oral challenge to canned mushrooms. Mushroom canning entails temperatures from 115–130 °C from 2 to 97 min, which has been thought to denature any heat labile proteins [11]. Passing an oral challenge to canned mushrooms with a positive raw mushroom skin test implies there is a possible reaction to a heat labile protein akin to PFAS patients tolerating cooked but not raw fruit/vegetables [6]. Positive SPT and IgE to well-known spore forming fungi and edible fungi of the phyla *Basidiomycete*s (*Agaricus bisporus*) and *Ascomycetes* (*Cladosporium herbarum, Alternaria alternata****,**** Penicillium notatum, Botrytis*, *Cephalosporium*, and *Pullaria*) suggests there is cross-reactivity occurring between these species causing sensitization and PFAS-like reaction with ingestion. Thus, we hypothesize the culprit protein to be a current unidentified heat-labile protein in the 47 kD or 67 kD range as suggested by Dauby et al. [8]. Future research utilizing the techniques of sodium dodecyl sulfate polyacrylamide gel electrophoresis with serum specific IgE immunoblotting followed by immunoblotting-inhibition testing to *Agaricus bisporus* and the patient’s mold sensitizations (*B. cinerea, C. gramineum*, *A. pullulans*, *A. alternata*, *C. herbarum*, and *P. notatum*) is required to verify cross reactivity.

It is plausible to postulate that the patient’s reaction could have been triggered by the presence of environmental aeroallergen spores on the outer surface of the mushroom pizza, considering that the conditions favorable for cultivating macrofungi are likely to support the growth of microfungi as well. However, we propose this explanation to be less probable, considering the patient’s medical history of experiencing seasonal and perennial rhinoconjunctivitis upon exposure to microfungi spores as well as positive SPT to multiple fresh mushrooms from different sources. It is noteworthy that the initial reaction occurred following the ingestion of mushroom-topped pizza, which theoretically would have led to the denaturation of heat-labile proteins. We hypothesize that the heterogeneous nature of vegetable thickness, cooking duration, and the presence of other food items that could disperse heat unevenly may account for this case; potentially allowing for the preservation of these heat labile cross-reactive proteins. Unfortunately, the canned mushroom species used for the oral challenge was not explicitly delineated. Nevertheless, *Agaricus bisporus* is the most widely cultivated and canned species in North America, while other varieties are generally identified with distinct labeling [12]. Given the prevalence of *Agaricus bisporus* and the observed morphological similarities between the canned mushrooms and *Agaricus bisporus*, we inferred the oral challenge was conducted with the same mushroom species employed in the SPT.

In conclusion, this case adds to the existing body of evidence supporting the relationship between sensitization to mold aeroallergens and allergy to edible fungi ingestion. However, knowledge of specific allergenic proteins that cause clinically relevant cross-reactions between molds and mushrooms is still limited. More research is required to understand the allergenic components that are involved and the specific species of mold and mushroom that are at risk of cross-reactivity.

## Data Availability

All data generated or analyzed during this study are included in this published article.

## Abbreviations

IgE: Immunoglobulin E
PFAS: Pollen food allergy syndrome
SPT: Skin prick test
